# Perceived barriers to physical activity among Nigerian stroke survivors

**DOI:** 10.11604/pamj.2015.21.274.6669

**Published:** 2015-08-11

**Authors:** Opeyemi Ayodiipo Idowu, Ade Fatai Adeniyi, Omoyemi Olubunmi Ogwumike, Henrietta Oluwafunmilola Fawole, Olayinka Akinrolie

**Affiliations:** 1 Department of Physiotherapy, School of Basic Medical Sciences, College of Medical Sciences, University of Benin, Benin-City, Nigeria; 2 Department of Physiotherapy, College of Medicine, University of Ibadan, Nigeria; 3 Centre for Research and Ageing, Faculty of Social and Human Sciences, University of Southampton, England

**Keywords:** Stroke survivors, physical activity, perceived barriers, Nigeria

## Abstract

**Introduction:**

Benefits of physical activity in the prevention and management of stroke are well documented in the literature. There is increasing evidence that stroke survivors in South-West Nigeria are physically inactive. Data on barriers to the achievement of the recommended physical activity levels including its differences along socio-demographic characteristics among stroke survivors in South-West Nigeria are needed.

**Methods:**

The Exercise Benefits and Barrier Scale and the International Physical Activity Questionnaire were administered on 121 stroke survivors to determine their perceived barriers to physical activity and physical activity levels respectively. Information on socio-demographic data and clinical variables were also collected.

**Results:**

The sample included 70.2% males, with majority of the participants reporting low physical activity levels (80.2%) and high perceived barriers (Mean = 48.13, SD = 7.88). The four most reported common barriers among stroke survivors were access to exercise facilities (95.0%), being embarrassed to exercise (94.2%), economic cost demands of exercise (94.2%) and notion that people in exercise clothes look funny (94.2%) respectively. There were no significant differences found in barriers to physical activity between gender (U= 1471.00, P= 0.74) and across each of: occupational status (H= 4.37, P = 0.22), age group (H= 0.82, P= 0.84) and educational levels (H= 4.56, P= 0.33). Significant difference however existed in perceived barriers across marital status categories (H = 12.87, P= 0.05)

**Conclusion:**

Stroke survivors indicated high perceived barriers to physical activity and these barriers were associated with marital status.

## Introduction

Stroke is a major cause of long-term disability and mortality worldwide. [1] When considered separately from other cardiovascular diseases, stroke is the 4^th^ leading cause of death in the United States of America (USA) [2, 3]. Potentially modifiable risk factors such as physical inactivity, overweight and obesity, diabetes mellitus, cigarette smoking, hypertension, abnormal blood lipids and lipoproteins have been implicated in the increasing incidence of stroke [4–9]. Addressing these factors through a combination of comprehensive lifestyle interventions and appropriate medical therapy is important in reducing the risk of stroke, lessening its severity and ensuring better long-term outcomes should an episode occur [10–12]. One such lifestyle intervention is physical activity (PA).

Regular PA is of immense benefit to stroke survivors. These include: improved balance and gait, improved cognition, enhanced arm recovery and function, improved cognitive function, increased self-efficacy levels, reduced fatigability and improved cardiovascular fitness [13, 14]. Worryingly, while the majority of stroke survivors have the ability to engage in higher levels of PA they rather chose not to [15, 16]. Further, participation in PA among this group of patients is substantially lower compared to either of their apparently healthy counterparts, [17–19] or people with other chronic illnesses [20–22]. Common reasons cited in the literature by stroke survivors for not adopting physically actives lifestyles include: costs associated with transportation to and registration with a fitness facility, lack of energy to exercise, stroke-related impairments, embarrassment and lack of knowledge about how and where to exercise [23]. Perceived barriers to PA have been posited to influence PA behavior change such that individuals who report less PA barriers are likely to become more physically active than those who report more barriers [20, 24]. Studies mostly from the developed world reported varied barriers to PA among stroke survivors [19, 24, 25]. To the best of our knowledge, there is no single study exclusively reporting barriers to PA among stroke survivors in Nigeria; there also seems to be limited published studies on perceived barriers reported by stroke survivors generally. Identifying and taking care of these barriers may help healthcare givers to better provide advice to stroke survivors on how to engage in, and achieve recommended levels of PA.

## Methods

### Participants

A convenient sample of 121 stroke survivors was included in the study. The recruitment was done over a period of three months. Participants were being managed exclusively for stroke at Ekiti State University Teaching Hospital, Ado, Ekiti State; University College Hospital, Ibadan, Oyo State; General Hospital, Ikere, Ekiti State and Ring Road State Hospital, Ibadan, Oyo State. They were recruited from the Neurology and Physiotherapy clinics of these hospitals. Ethical approval was obtained from the University of Ibadan/ University College Hospital Research Ethics Committee (UI/EC/10/0175) and a written informed consent was obtained from each participant before data collection. Inclusion criteria included being managed exclusively for stroke, not presenting with additional disabling conditions such as blindness and amputations and willingness to fill the informed consent forms.

### Instruments

*Socio-demographic Data*: A structured questionnaire was used to obtain socio-demographic data (age, gender, marital status, level of education and occupational status) from participants. Occupational status was categorized as unemployed, self-employed, employed by government or corporate bodies and retirees. Participants’ age was grouped into 31-40 years, 41-50 years, 51-60 years and above 60 years. Marital status was classified as singled, married, divorced and widowed. Educational level was classified into five groups: none, primary, polytechnic, college of education and university. Clinical information such as duration of stroke and side of affectation was extracted from patients’ case notes.

*Physical activity measure*: The English and Yoruba versions (translated from the English version) of the short, seven-item, last week version of the International Physical Activity Questionnaire (IPAQ) was used to measure the self-reported physical activity of participants [19, 26]. The IPAQ was administered and scored on stipulated criteria [26]. The score was calculated using the sum of walking MET-minutes/ week, moderate MET-minutes/ week and vigorous MET-minutes/ week. The IPAQ defines moderate activity as an activity, performed for at least 10 minutes, that produces an increase in breathing and heart rates and causes sweating. Vigorous activities was defined as those activities performed for at least 10 minutes which produces even greater increase in breathing, heart rate, and sweating. Walking was defined as the time spent walking in the last 7 days. Stroke survivors who reported five or more days of any combination of walking, moderate-intensity or vigorous-intensity activities achieving a minimum of at least 600 MET-min/weeks were adjudged to have moderate PA levels while those who reported no activity or less than the criteria of moderate PA were considered to have low PA levels. Similarly, individuals who reported at least 3000 MET-minutes/week of any combination of walking, moderate-or vigorous-intensity activities accumulating on 3 or more days were considered to have high PA levels. A study by Craig et al reported a high test-retest reliability of the short form of the IPAQ (spearman's Rho = 0.75, N= 292) [27]. Adeniyi and colleagues reported a similar test-retest reliability of the Yoruba version of the IPAQ-SF (ICC = 0.86, 95% CI = 0.82-0.94) [19].

*Barriers to Physical activity Measure*: The Barrier component of the Exercise Benefits and Barrier Scale (EBSS), a self-administered questionnaire developed by Sechrist et al, was used to assess the barriers to physical activity of stroke survivors [28]. The barrier component of the EBBS consists of 14 items rated on a 4-point Likert-type scale. The 14 items are further categorized into four subscales: exercise milieu (items 1-6); time expenditure (items 7-9); physical exertion (items 10-12); and family discouragement (items 13-14). It has been found to have a high internal consistency (0.87) and test re-test reliability (0.77). It has been used in several studies [28–30]. The minimum score for the barrier scale is 14 which show less perceived barriers to physical activity while the maximum score is 56.

### Data Analysis

Statistical analysis was performed using IBM SPSS statistics 19 for Windows (SPSS Inc, Chicago, IL, USA). Descriptive statistics were presented as means, frequencies and percentages. Mann-Whitney U test was used to compare the perceived barriers to PA between male and female stroke survivors. Kruskal Wallis test was used to compare perceived barriers across other socio-demographic variables such as occupational status, age group, marital status and educational level. Independent t test was used to compare the PA score and duration of diagnosis of stroke between male and female participants. P < 0.05 was set as the significant value for all the tests.

### Results

Out of the 133 participants that were recruited for the study, 12 were excluded due to refusal to give informed consent and or incomplete data leaving a total number of 121 stroke survivors (70.25% males vs. 29.75% females) who were included in the statistical analysis. Their socio-demographic data are shown in Table 1. The mean duration of stroke of the stroke survivors was 4.26 ± 3.14 years while 36.36% were between the age group of 51-60 years.. Majority of participants were physically inactive (80.2%) while the remaining participants reported moderate PA levels. Examined by gender, women reported a slightly higher level of physical inactivity (80.6%) compared to men (80.0%); however this did not differ significantly (t= 1.71, P = 0.86). Also, there was no significant difference in the duration of stroke between gender (t = 1.60. P = 0.11).

**Table 1:** Socio-demographic characteristics of participants

Variable	Frequency	Percentage
**Gender**		
Male	85	70.25
Female	36	29.75
**Occupational status**		
Self employed	52	42.98
Employed by Government/ Corporate bodies	32	26.45
Retirees	23	19.00
Unemployed	14	11.57
**Age Group**		
31-40	25	20.66
41-50	34	28.10
51-60	44	36.36
Above 60	18	14.88
**Marital Status**		
Single	4	3.30
Married	85	70.25
Divorced	14	11.57
Widowed	18	14.88
**Educational level**		
None	18	14.90
Primary	23	19.00
Polytechnic	25	20.66
College of Education	29	23.97
University	26	21.49

Regarding barriers to PA, 100% of the participants, regardless of age group and gender, reported at least one barrier that prevented or reduced their participation in recommended PA. The participants had a mean perceived barrier score of 48.13 (SD = 7.88). For the sake of analysis, the four possible responses on the EBSS (strongly agree, agree, disagree, strongly disagree) were dichotomized (agree or disagree). Although not significant, more female participants’ seemed to have indicated perceived barriers in the individual barrier items (9 out of 15). Male participants reported more often perceived barriers in the following: “access to exercise facilities”, “exercise facilities not having convenient schedules”, “exercise taking too much time”, “lack of encouragement from spouse” and lack of encouragement from family” (Table 2). There were no significant differences when perceived barrier to PA scores were compared between gender (U= 1471.00, p= 0.74) and across each of occupational status (H= 4.37, p = 0.22), age group (H= 0.82, p= 0.84) and educational level (H= 4.56, p= 0.33). However, there was a significant difference (H = 12.87, p= 0.05) when perceived barriers were compared across marital status categories (Table 3).

**Table 2:** Self-reported barriers to physical activity as reported by stroke survivors (N = 121)

Barrier	Males (85) N (%)	Females (36) N (%)	χ^2^	P
Places for me to exercise are too far away	81 (95.3)	34 (94.4)	0.00	1.00
I am too embarrassed to exercise	80 (94.1)	34 (94.4)	0.00	1.00
It costs too much money to exercise	80 (94.1)	34 (94.4)	0.00	1.00
Exercise facilities do not have convenient schedules for me	74 (87.1)	31(86.1)	0.00	1.00
I think people in exercise clothes look funny	80 (94.1)	34 (94.4)	0.00	1.00
There are too few places for me to exercise	75 (88.2)	32 (88.9)	0.00	1.00
Exercise takes too much time from family relationships	74 (87.1)	33 (91.7)	0.17	0.68
Exercise takes too much time from my family responsibilities	67 (78.8)	30 (83.3)	0.32	0.57
Exercising takes too much of my time	70 (82.4)	29 (80.6)	0.05	0.82
Exercise tires me	71 (83.5)	32 (88.9)	0.57	0.45
I am fatigued by exercise	71 (83.5)	31 (86.1)	0.13	0.72
Exercise is hard work for me	73 (85.9)	31 (86.1)	0.00	0.97
My spouse (or significant other) does not encourage exercising	76 (89.4)	36 (88.9)	0.00	1.00
My family (or significant other) does not encourage exercising	72 (84.7)	29 (80.6)	0.32	0.57

**Table 3:** Test of significant difference in perceived barriers to physical activity scores as defined by socio-demographic variables of stroke survivors

Variable		Mean Rank	Statistical value	P- value
**Gender**	Male	60.31	1471.00^a^	0.74
	Female	62.64		
**Occupational status**	Self-employed	53.47	4.37 ^b^	0.22
	Employed by Government/ Corporate bodies	65.25		
	Retiree	67.15		
	Unemployed	69.14		
**Age group**	31-40	66.22		
	41-50	58.09	0.82 ^b^	0.84
	51-60	60.16		
	Above 60	61.31		
**Marital status**	Single	111.13		
	Married	62.39	12.87 ^b^	0.05*
	Divorced	41.39		
	Widowed	58.56		
**Educational level**	None	53.17		
	Primary	57.3	4.56 ^b^	0.33
	Polytechnic	62.2		
	College of Education	57.1		
	University	72.88		

### Discussion

This study explored the perceived barriers to PA among a group of stroke survivors from Nigeria. Majority of participants in this study were observed to be physically inactive. This is in line with other studies documenting low PA levels among stroke survivors [19, 31]. In third world countries, major family participation in hospital and home care is essential for survival and quality of life after stroke [32]. However, such assistance may be overbearing and counterproductive, especially when the patient could actually muster some ability to engage in PA or activities of daily living. Leading a physically inactive and sedentary lifestyle could further worsen patient's condition [14]. Patients should therefore be allowed and encouraged to engage in free-living activity at home to prevent recurrence and enhance general health status. Also there may be need to adapt the living environment, work environment and even the community at large to facilitate the engagement of stroke survivors in sufficient PA.

The overwhelming majority of participants reported that both intrinsic and extrinsic factors such as access to exercise facilities, being embarrassed to exercise, economic cost demands of exercise, notion that people in exercise clothes look funny and being fatigued by exercise amidst others limited their desire to take part in exercise. In comparison with the study of McDonnell et al, [24] factors such as cost demands of exercise and access to facilities which were reported as major barriers in our study scored low. There are two major differences between both studies. Firstly, the stroke survivors in our study, with four-year mean duration of stroke were receiving medical attention on outpatient basis while the other study was conducted among inpatients that had just had an episode of stroke not more than a month. While our study included PA months and years after stroke occurrence, theirs focused on pre-morbid PA status. Secondly, the instrument used for assessing barriers to PA differed. In the present study, the EBBS, [28] was used in contrast with the barriers to Physical Activity in Disability Survey [18] used by McDonnell et al [24]. These could have accounted for the differences in results. Rimmer et al found that the costs of joining a fitness facility and transportation to the facility are major barriers reported by stroke survivors [18]. The same was also reported in our study. It is not surprising that economic implication of joining a fitness facility is a major barrier among participants in this part of the world. Due to increased activity limitation and increased participation restriction resulting in reduced cardiorespiratory fitness and eventually reduced functional capacity, [33] stroke survivors often lose their jobs prematurely. Thus they may have to rely solely on their family for support [34]. This places financial burden on the support providers and implies prioritizing on health spending. As a result, many stroke survivors are usually unable to afford the services of an exercise expert (such as a physiotherapist) post-discharge; thus relying on few outpatient sessions offered to them at public hospitals which is grossly inadequate to meet their health needs [35]. On the part of the health care givers, counselling of stroke survivors on affordable and enjoyable ways to engage in PA is important. For example, walking is a very cheap and rewarding way to engage in PA, [36] stroke survivors should therefore be encouraged to walk regularly.

Our results showed no significant differences in barriers to PA when examined by most socio-demographic factors such as gender, occupational status, age group, and educational level. However, there was a statistical significant difference in perceived barriers to PA across marital status categories with increased perceived barriers reported more by those who were single. Most researches on barriers to PA among stroke survivors did not explore associations between barriers and socio-demographic characteristics [24, 25]. Rimmer et al. reported an association between barriers and income earned with lower income group identifying cost and lack of transportation as primary barriers to PA [18]. It is therefore unlikely that there would be differences in perceived barriers when examined across socio-demographic factors. The reason for the difference in perceived barriers that existed across marital status categories in this study is unknown. However, several other factors such as social support and income may have played a role in this association. It is important to provide adequate support system to enable patients adequately take part in PA. Also, barriers such as costs and lack of transportation should ideally be removed and stroke survivors should have access to exercise guidance delivered by instructors with suitable knowledge and training.

An important strength of this study is that it has provided information on perceived barriers to PA among stroke survivors from a Nigeria perspective which was hitherto scarce. Literature on PA have recommended the use of accelerometers when measuring PA levels because it is a direct measure and provides more reliable information. However, accelerometers are costly and may not be affordable especially in large scale studies. Therefore we used the IPAQ which has also been found to be valid, despite the often reported limitations. Another limitation of this study is that it was conducted in two states in South West, Nigeria, thus generalization of the results is limited.

## Conclusion

Stroke survivors indicated high perceived barriers to PA. However, only marital status out of all the socio-demographic factors explored was associated with PA.
